# HLA Expression in Relation to HLA Type in Classic Hodgkin Lymphoma Patients

**DOI:** 10.3390/cancers13225833

**Published:** 2021-11-20

**Authors:** Geok Wee Tan, Peijia Jiang, Ilja M. Nolte, Kushi Kushekhar, Rianne N. Veenstra, Bouke G. Hepkema, Ruth F. Jarrett, Anke van den Berg, Arjan Diepstra

**Affiliations:** 1 Department of Pathology and Medical Biology, University of Groningen, University Medical Center Groningen, 9700 RB Groningen, The Netherlands; g.w.tan@umcg.nl (G.W.T.); p.jiang@umcg.nl (P.J.); kushekhar@hotmail.com (K.K.); rianneveenstra@icloud.com (R.N.V.); a.van.den.berg01@umcg.nl (A.v.d.B.); 2 Molecular Pathology Unit, Cancer Research Centre, Institute for Medical Research, National Institutes of Health, Ministry of Health, Shah Alam 40170, Selangor, Malaysia; 3 Department of Laboratory Medicine, Shenyang Huanggu National Defence Hospital, Shenyang 110032, China; 4 Department of Epidemiology, University of Groningen, University Medical Center Groningen, 9700 RB Groningen, The Netherlands; i.m.nolte@umcg.nl; 5 Department of Laboratory Medicine, University of Groningen, University Medical Center Groningen, 9700 RB Groningen, The Netherlands; b.g.hepkema@umcg.nl; 6 Institute of Infection, Immunity and Inflammation, College of Medical, Veterinary and Life Sciences, MRC-University of Glasgow Centre for Virus Research, Glasgow G61 1QH, UK; Ruth.jarrett@glasgow.ac.uk

**Keywords:** cHL, susceptibility, HLA expression, EBV

## Abstract

**Simple Summary:**

Classic Hodgkin lymphoma (cHL) is a B-cell malignancy with involvement of Epstein–Barr virus (EBV) in about 30% of the European population. The risk to develop cHL is strongly linked to genetic variants in the human leukocyte antigen (HLA) genomic region and to certain HLA alleles. This may be caused by the function of HLA alleles, or by genetic linkage to non-HLA genes. HLA can present EBV-derived and tumour-cell specific antigens and this may lead to anti-tumour immune responses. However, the tumour cells downregulate HLA expression in a proportion of the cases, which may result in immune escape. In this study, we tested whether the loss of HLA expression is related to the presence of certain protective HLA alleles. We found that loss and retention of HLA expression is indeed associated with presence of known susceptibility HLA alleles. These findings suggest that HLA itself is involved in development of cHL.

**Abstract:**

Several human leukocyte antigen (HLA) alleles are strongly associated with susceptibility to classic Hodgkin lymphoma (cHL), also in subgroups stratified for presence of the Epstein–Barr virus (EBV). We tested the hypothesis that the pressure on cHL tumour cells to lose HLA expression is associated with HLA susceptibility alleles. A meta-analysis was carried out to identify consistent protective and risk HLA alleles in a combined cohort of 839 cHL patients from the Netherlands and the United Kingdom. Tumour cell HLA expression was studied in 338 cHL cases from these two cohorts and correlated to the presence of specific susceptibility HLA alleles. Carriers of the HLA-DRB1*07 protective allele frequently lost HLA class II expression in cHL overall. Patients carrying the HLA-DRB1*15/16 (DR2) risk allele retained HLA class II expression in EBV− cHL and patients with the HLA-B*37 risk allele retained HLA class I expression more frequently than non-carriers in EBV+ cHL. The other susceptibility alleles showed no significant differences in expression. Thus, HLA expression by tumour cells is associated with a subset of the protective and risk alleles. This strongly suggests that HLA associations in cHL are related to peptide binding capacities of specific HLA alleles.

## 1. Introduction

Classic Hodgkin lymphoma (cHL) is a B-cell malignancy characterised by an abundant infiltrate of lymphocytes surrounding a minority of tumour cells known as Hodgkin and Reed-Sternberg (HRS) cells. CHL is categorised into four different histological subtypes: nodular sclerosis (NS); mixed cellularity (MC); lymphocyte depleted (LD); and lymphocyte rich (LR). The incidence of cHL follows a bimodal distribution with peaks in young adults and individuals aged > 55 [1]. Prevalence of Epstein–Barr virus (EBV) in HRS cells varies between different parts of the world, with approximately 30% of Western European cHL cases being EBV+ [2]. The percentage of EBV+ cHL cases is higher in the MC subtype group and in male patients.

In the general population, primary EBV infection is ubiquitous and the virus persists in a latent stage in a small pool of B-cells throughout life [3]. Lifelong T-cell mediated immune responses restrict the number of EBV infected B-cells and prevent malignant transformation by recognition of EBV-derived antigenic peptides presented in the context of the human leukocyte antigen (HLA) [4]. B-cells constitutively express HLA class I and HLA class II on the cell surface, and at the time of diagnosis, HRS cells can interact extensively with CD4+ T cells by HLA class II and immunological synapse formation [5]. However, in ~65% of cases the HRS cells lack cell surface expression of HLA class I and this is observed most often in EBV− cHL [6]. In addition, lack of HLA class II membranous expression is observed in ~40% of cHL cases, with a somewhat higher incidence in EBV− compared to EBV+ cHL [6,7]. In many cHL cases, HRS precursor cells most likely circumvent immune recognition by downregulating HLA expression, which prevents presentation of tumour cell derived antigenic peptides and subsequent recognition by T cells.

Further evidence indicating an important role of HLA type in early cHL development comes from HLA typing and genetic association studies in both sporadic and familial cHL cases [8,9,10]. Genome-wide association studies (GWAS) have consistently shown dominant associations of the HLA region with cHL overall and EBV-stratified subgroups [11,12,13,14]. Currently known associations with HLA alleles include HLA-B*51/52 (*B5*) as a risk and HLA-DRB1*07 as a protective allele for cHL overall; HLA-A*01, HLA-B*37, and HLA-DRB1*10 as risk and HLA-A*02 as protective alleles for EBV+ cHL; and HLA-DRB1*11/12 (*DR5*) and HLA-DRB1*15/16 (*DR2*) as risk alleles for EBV− cHL [15,16,17,18]. It is well known that the repertoire of antigenic peptides that can be presented depends on the HLA alleles [19]. Accordingly, these associations may reflect a differential capacity of HLA alleles to present tumour cell-derived or EBV-derived antigenic peptides to immune cells. Alternatively, the HLA associations may be caused by linkage to any of the non-HLA genes in this genomic locus. It is extremely difficult to discriminate between these two because the HLA region is very polymorphic.

In this study, we hypothesised that HRS cells in patients who carry protective HLA alleles downregulate expression of HLA to escape from effective antitumour immune responses. Presence of HLA risk alleles may diminish this selective pressure. To elucidate this, we determined whether HLA class I and HLA class II expression by HRS cells at the time of diagnosis is associated with HLA alleles known to confer protection or risk for the development of cHL overall and in EBV-stratified subgroups.

## 2. Results

### 2.1. Patient Characteristics and HLA Expression

**Figure 1:**
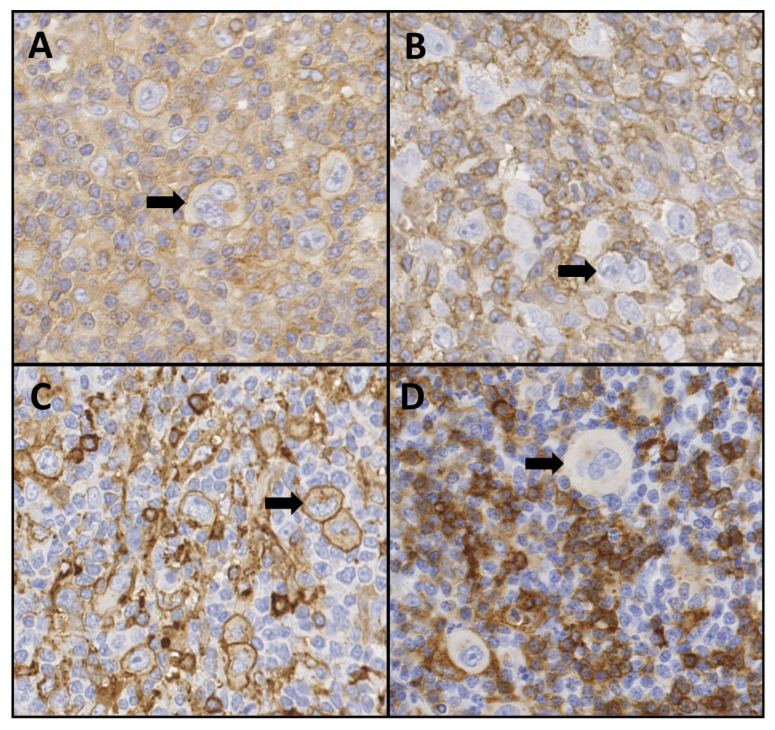
Representative images of immunohistochemistry staining for B2M and HLA class II in classic Hodgkin lymphoma tissue. Scoring is based on staining of the cell membrane of Hodgkin and Reed Sternberg cells. (**A**) B2M positive, (**B**) B2M negative, (**C**) HLA class II positive, (**D**) HLA class II negative. Arrows indicate representative HRS cells. 40× magnification.

The median age of cHL patients (*n* = 338) was 32 years (range 12 to 78), with slightly more males (52.4%). NSHL was the most common subtype accounting for 77.2% of all cases. Presence of EBV was observed in 25.4% of the cases (Table 1). Tumour cell immunohistochemistry staining for HLA class I and HLA class II was performed if not already available (representative images shown in Figure 1). The expression status was not evaluable in 34 (10.1%) cases for HLA class I and 27 (8.0%) cases for HLA class II due to a low number of tumour cells in the tissue or suboptimal quality of staining. HLA class I was downregulated in 72.0% (219/304) of cHL cases. Loss of HLA class I expression was significantly more frequent in females (79.6% vs. 65.0%, *p* = 0.0045), EBV− cases (81.9% vs. 42.1%, *p* < 0.00001), NS subtype in comparison to MC subtype (77.6% vs. 43.6%, *p* < 0.00001), and at younger age (median age 29 vs. 37 years, *p* = 0.0032) (Table 1). These differences are driven by EBV status as this was the only variable to show a significant association (*p* < 0.00001) in a multivariable analysis including gender, subtype, and age. Downregulation of HLA class II was observed in 40.8% (127/311) of all cHL cases. Loss of HLA class II was observed more frequently in older cHL patients (median age 36 vs. 30 years, *p* = 0.0067) as compared to younger cHL patients (Table 1).

### 2.2. Identification of HLA Susceptibility Alleles in the Combined Dutch and UK cHL Cohorts

Meta-analysis confirmed previously observed associations of HLA-DRB1*04 and HLA-DRB1*07 for cHL overall, HLA-B*07, HLA-DRB1*15/16, and HLA-DRB1*11/12 for EBV− cHL, and five known associations, i.e., HLA-A*01, HLA-A*02, HLA-B*08, HLA-B*37, and HLA-DRB1*03, for EBV+ cHL (Table 2 and Supplementary Figure S1). Associations in the individual cohorts showed the same risk pattern as observed in the meta-analysis. In addition, we observed a significant protective association with cHL overall for HLA-B*44/45, which did not reach significance in the two separate cohorts but did show similar odds ratio (OR) patterns (Supplementary Figure S1). This led to inclusion of a total of three HLA alleles for cHL overall, three alleles for EBV− cHL, and five alleles for EBV+ cHL, for the subsequent downstream association analyses with HLA class I and class II expression.

### 2.3. HLA Expression and Susceptibility in cHL Patients

**Figure 2:**
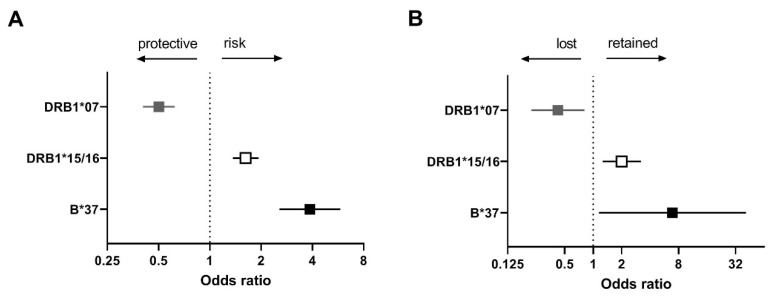
Association between cHL-associated HLA alleles and HLA expression. The odds ratios (squares) and 95% confidence intervals (whiskers) of cHL-associated HLA alleles (**A**) and loss or retention of HLA expression (**B**) in cHL overall (grey), EBV− cHL (white), and EBV+ cHL (black). The black arrows above the forest plot indicate protective or risk effects of cHL (**A**) and loss or retention of expression of the corresponding HLA class (**B**).

HLA-DRB1*07 protective allele carriers more often lost HLA class II expression than non-carriers in cHL overall (60% vs. 40%, *p* = 0.015) consistent with our hypothesis (Figure 2A,B). HLA-B*44/45 and HLA-DRB1*04 were not significant. 

EBV− cHL patients carrying the HLA-DRB1*15/16 risk allele more frequently retained HLA class II expression (69% vs. 52%, *p* = 0.007), consistent with our hypothesis (Figure 2A,B). No significant differences were observed for patients carrying the risk alleles HLA-B*07 and HLA-DRB1*11/12. 

EBV+ cHL patients carrying the HLA-B*37 risk allele more frequently retained HLA class I expression (89% vs. 54%, *p* = 0.038) (Figure 2A,B). The remaining alleles were not significant, but the HLA staining patterns for HLA-A*01, HLA-A*02, and HLA-DRB1*03 showed a slight trend consistent with the hypothesis. 

## 3. Discussion

In this study, we tested the hypothesis that HRS cells in carriers of protective HLA alleles have a higher selective pressure to lose HLA expression, while there is no pressure to lose HLA expression in HLA risk allele carriers. Significant associations were found for one of the protective alleles and two of the risk alleles. Patients carrying the protective HLA-DRB1*07 allele more frequently lost HLA class II expression than non-carriers in cHL overall. In addition, EBV− cHL patients carrying the HLA-DRB1*15/16 (*DR2*) risk allele more frequently retained HLA class II expression than non-carriers and EBV+ cHL patients carrying the HLA-B*37 risk allele more frequently retained HLA expression compared to non-carriers. 

We combined cHL cases and controls from a Dutch and a UK cohort and performed a meta-analysis to identify shared susceptibility alleles. This revealed significant associations for all the alleles previously reported to be associated with either of these cohorts [17,18]. In addition, we identified one novel cHL-associated HLA allele, i.e., HLA-B*44/45. As expected, this protective allele showed corresponding trends in both original studies.

We determined membranous expression of HLA class I and HLA class II on HRS cells by immunohistochemistry, reflecting expression of all alleles. It should be noted that individual HLA alleles can mostly not be detected in formalin-fixed paraffin-embedded tissue because suitable allele specific antibodies do not exist. However, downregulation of only one specific HLA gene is very rare in malignancies in general, while loss of expression of all class I or class II HLA alleles is a ubiquitous immune escape mechanism, also in lymphomas. Mechanisms of downregulation include somatic mutations in the *b2-microglobulin* (*B2M*) gene, that is essential for stable membranous expression of HLA class I [20,21]. In addition, epigenetic modifications of the HLA class I gene locus can also lead to downregulation of HLA class I as shown in multiple cancer types [22]. The mechanisms that cause loss of HLA class II expression in B-cells have not been fully elucidated, but have been attributed in part to partial plasma cell differentiation and in part to mutations in genes of the HLA class II processing pathway [23,24]. Other mechanisms include deletions of the entire HLA locus as shown in diffuse large B-cell lymphoma [25,26].

In our patient cohort, membranous expression of HLA class I on HRS cells was lost in 72% of the cases, while HLA class II was lost in 41% of the cases. Loss of both HLA class I and class II was observed in 32% of the cases. These frequencies of HLA loss are similar to previously published results [27,28,29]. Downregulation of HLA class I expression was significantly more frequent in younger and female patients, and in EBV− NS subtype cases. These differences are probably driven by the well-known association between loss of HLA class I expression and negative EBV status. Females are more likely to develop EBV− cHL than males [30] and EBV− cases are more common in the NS subtype, which constitutes the majority of young adult cases. Downregulation of HLA class II was more frequent in older patients and was not related to other factors. 

We observed an expression pattern consistent with our hypothesis, i.e., loss of HLA expression for one protective allele and retention of HLA expression for two risk alleles. The protective HLA-DRB1*07 allele was commonly lost in cHL overall. This suggests that HLA-DRB1*07 carriers are likely to present tumour cell derived antigenic peptides, and that downregulation of HLA expression in HRS cells is required to overcome CD4+ T cell orchestrated anti-tumour responses. The HLA class II allele, HLA-DRB1*15/16, is a risk allele for EBV− cHL and is associated with retained HLA class II expression. This fits with our hypothesis assuming that there is no pressure for HRS cells to lose expression in risk allele carriers. For the HLA-B*37 risk allele in EBV+ cHL, HLA class I expression was preferentially retained. EBV specific immune responses in the context of HLA-B*37 have never been found, so there may be no or little selective pressure to downregulate HLA class I expression. In addition, where HRS cells are not susceptible to cytotoxic T cell responses, retention of HLA class I expression may favour escape from natural killer cell mediated anti-tumour responses. For the EBV+ cHL subgroup the HLA-A*01 risk allele and the HLA-A*02 protective allele also showed a pattern consistent with our hypothesis, although this association was not statistically significant. The association of HLA-A*01 with an increased risk of developing EBV+ cHL has been linked to the incapacity to present EBV-derived peptides, prohibiting induction of effective CD8+ T cell responses. In contrast, the HLA-A*02 can present EBV-derived antigenic peptides to CD8+ T cells and protects against EBV+ cHL [31,32,33,34].

For six other susceptibility or protective alleles, no significant differences in HLA expression were found (HLA-B*44/45 and HLA-DRB1*04 in overall, HLA-B*07 and HLA-DRB1*11/12 in EBV−, and HLA-B*08 and HLA-DRB1*03 in EBV+ cHL). These findings suggest that the mechanism of HLA retention/downregulation is more complex than suggested in our hypothesis. One confounding factor is that HRS cells can express multiple protective and/or risk alleles, i.e., up to 18 different HLA class I and class II alleles. Linkage disequilibrium (LD) between alleles, which is extensive in the HLA region, may couple risk alleles to protective alleles, potentially leading to contradictory findings when single alleles are being studied. For example, the HLA-B*07 risk allele is in strong LD with the protective HLA-A*02 allele. Unfortunately, our sample size did not allow meaningful testing of combinations of HLA alleles because of lack of statistical power. Another confounding factor is that polymorphisms in other components of the antigen presenting pathway, like *ERAP1*, *ERAP2*, *TAP1*, and *TAP2* [35,36,37], may affect antigen presentation and disrupt associations. In addition, 2-digit HLA typing defines allele groups that are highly similar but not identical, which might result in some heterogeneity within groups and therefore mask potential associations. Moreover, during the development of cHL, acquired recurrent and unique mutations in HRS cells can act as neo-antigens and increase the strength of anti-tumour immune responses over time. Loss of HLA expression can only be measured at diagnosis, but the timing of this loss may be different between cases. It is important to realise that the function and biology of HLA alleles is highly similar, but the spectrum of antigenic peptides they present is very diverse. Therefore, although the aetiology is complex, the fact that multiple HLA alleles affect HLA retention/downregulation strongly supports a role for antigen presentation. However, we cannot exclude that part of the HLA associations are driven by linkage to non-HLA genes.

## 4. Materials and Methods

### 4.1. Study Outline

For this study, we combined cHL patients derived from a Dutch (*n* = 254) and a United Kingdom (UK) cohort (*n* = 84), for which both HLA typing data and HLA staining data or tissue sections to determine HLA expression were available [6,7,17,18]. As HLA allele associations were slightly different between both cohorts, we first performed a meta-analysis of a Dutch and UK association study of the above-mentioned cases with 7554 Dutch and 347 UK controls, respectively, to establish common risk and protective alleles using the previously published data. Allele frequencies in HLA expression positive and negative subgroups were compared to identify potential associations in cHL overall and EBV-stratified subgroups.

### 4.2. Patients

Patient characteristics (*n* = 338) are described in Table 1. All patients gave written informed consent, and the protocols were approved by the medical ethics board of the University Medical Centre, Groningen, the Netherlands and by NHS Research Ethics Committees, in the UK. HLA typing data were obtained previously using a PCR based sequence-specific oligonucleotide probe hybridisation (SSOP) [17,18]. For the Dutch patients, 1st field typing was done, while 2nd field typing was performed for the UK cases. The 2nd field typing of the UK cohort was converted to a 1st field typing to allow combination of both cohorts. EBV positivity was determined previously by EBV encoded small RNA (EBER) in situ hybridisation or LMP1 immunohistochemistry. 

### 4.3. HLA Class I and Class II Expression

For 121 out of 254 Dutch cHL patients, results of HLA class I and HLA class II immunohistochemistry were available from our previous study [7]. For the remaining Dutch and all other UK cases, formalin-fixed paraffin-embedded tissue sections were stained and scored following the same procedures using the polyclonal rabbit anti-human B2M antibody (DAKO, Glostrup, Denmark) to determine membranous HLA class I staining. Of note, in our experience the B2M antibody reflects expression of classical HLA class I genes HLA-A, B, and C very well [38]. Although B2M protein also associates to non-classical HLA class I molecules HLA-E and HLA-G, this does not interfere since both are only expressed at low levels and HLA-E expression is concordant with expression of the classical HLA class I proteins [38,39]. The CR3/43 monoclonal antibody (DAKO, Glostrup, Denmark) binding specifically to monomorphic epitopes in the beta-chain of HLA-DP, HLA-DQ, and HLA-DR (the complete set of HLA class II antigen presenting proteins) was used to determine HLA class II membranous expression. Sections were only scored when the internal positive control (lymphocytes) showed consistent staining and when at least 50 HRS cells were present for evaluation. Membranous HLA expression of HRS cells was scored positive if there was accentuation relative to the surrounding lymphocytes or when present in between adjacent HRS cells. Lack of membranous staining between adjacent HRS cells was scored as negative. In cases with both positive and negative staining of HRS cells, the predominant staining pattern (>50%) determined the score.

### 4.4. Meta-Analysis of Susceptibility Alleles

A Mantel–Haenszel meta-analysis was performed to identify consistent HLA susceptibility alleles of the Dutch (*n* = 337) and UK (*n* = 502) cohorts for cHL overall and for EBV-stratified subgroups using previously published data (Supplementary Table S1) [17,18]. EBV status was available for 812 patients (232 (27.7%) EBV+ and 580 (69.1%) EBV− cHL). HLA alleles with frequencies > 10% and a *p*-value < 0.1 in either cHL overall or EBV-stratified subgroups in the original Dutch or UK cHL susceptibility studies [17,18] were included in our meta-analysis. 

For the meta-analysis we calculated ORs, 95% confidence intervals (CIs), and *p*-values to define consistent susceptibility alleles for cHL-overall or EBV-stratified subgroups. Allele associations with a *p*-value < 0.01 in the meta-analysis and a pattern consistent with the patterns observed in both original studies were selected for the subsequent association study with HLA expression. 

### 4.5. HLA Expression Analyses

Carrier frequencies of alleles that were associated with cHL in the combined cohort and had a frequency of at least 10% in the combined cohort, i.e., either in the total or in EBV-stratified subgroups, were compared between groups with and without membranous HLA expression in cHL overall and in EBV stratified subgroups. Association analyses were performed between HLA class I expression and HLA-A, HLA-B, and HLA-C susceptibility alleles and between HLA class II expression and HLA-DR susceptibility alleles. Differences in HLA expression were assessed by one-sided Chi-square or Fisher’s exact tests. We considered *p* < 0.05 to be significant.

## 5. Conclusions

In conclusion, retention or downregulation of HLA expression in cHL is associated with the HLA-DRB1*07 protective allele in cHL overall, the HLA-DRB1*15/16 risk allele in EBV− cHL and the HLA-B*37 risk allele in EBV+ cHL. Our findings strengthen the notion that HLA associations in cHL are at least partly based on the specificity of specific HLA types to bind and present antigenic peptides to T cells.

## Figures and Tables

**Table 1:** Patient characteristics in total and HRS cell HLA expression status stratified subgroups.

cHL Patient	Total	HLA I+	HLA I−	*p*-Value	HLA II+	HLA II−	*p*-Value
	(*n* = 338)	(*n* = 85)	(*n* = 219)		(*n* = 184)	(*n* = 127)	
Gender, *n* (%)							
Male	177 (52.4)	55 (35.0)	102 (65.0)	0.0045 ^a^	96 (58.9)	67 (41.1)	ns ^a^
Female	161 (47.6)	30 (20.4)	117 (79.6)		88 (59.5)	60 (40.5)	
Age							
Median (range)	32 (12–78)	37 (16–72)	29 (12–78)	0.0032 ^b^	30 (14–78)	36 (15–70)	0.0067 ^b^
EBV status, *n* (%)							
EBV positive	86 (25.4)	44 (57.9)	32 (42.1)	<0.00001 ^a^	43 (58.1)	31 (41.9)	ns ^a^
EBV negative	250 (74.0)	41 (18.1)	185 (81.9)		140 (59.3)	96 (40.7)	
NA	2 (0.6)	0 (0)	2 (100)		1 (100)	0 (0)	
Subtype, *n* (%)							
NS	261 (77.2)	53 (22.4)	184 (77.6)	0.00016 ^b^	147 (62.3)	89 (37.7)	ns ^a^
MC	43 (12.7)	22 (56.4)	17 (43.6)		25 (59.5)	17 (40.5)	
LR	7 (2.1)	1 (16.7)	5 (83.3)		2 (28.6)	5 (71.4)	
LD	1 (0.3)	0 (0)	1 (100)		0 (0)	1 (100)	
NOS	26 (7.7)	9 (42.9)	12 (57.1)		10 (40.0)	15 (60.0)	

^a^ Chi-square test. ^b^ Mann–Whitney test. Abbreviations: NS, nodular sclerosis; MC, mixed cellularity; LR, lymphocyte rich; LD, lymphocyte depletion; NOS, not otherwise specified; NA, not available; ns, not significant.

**Table 2:** Meta-analysis of cHL-associated HLA alleles.

cHL	HLA Type	OR	*p*-Value
Overall	B*44/45	*0.68*	*1.4 × 10^−4^*
	DRB1*04	0.59	1.8 × 10^−6^
	DRB1*07	0.50	1.3 × 10^−7^
EBV−	B*07	1.49	1.9 × 10^−4^
	DRB1*15/16	1.62	5.0 × 10^−6^
	DRB1*11/12	1.38	8.7 × 10^−3^
EBV+	A*01	2.88	5.1 × 10^−12^
	A*02	0.52	2.7 × 10^−5^
	B*08	2.12	2.0 × 10^−6^
	B*37	3.87	6.4 × 10^−8^
	DRB1*03	1.70	9.7 × 10^−4^

The association shown in italic was not significant in the individual studies, but ORs show a similar trend in both cohorts.

## Data Availability

The data that support the findings of this study are available from the corresponding author upon reasonable request.

## Supplementary Materials

The following are available online at https://www.mdpi.com/article/10.3390/cancers13225833/s1, Figure S1: Forest plots of significant cHL susceptibility HLA alleles, Table S1: Number of subjects in meta-analysis and HLA expression analysis, Table S2: Frequencies of HLA alleles in patients and controls.

## References

[1] Engert A., Younes A. (2020). Hodgkin Lymphoma A Comprehensive Overview.

[2] Deacon E.M., Pallesen G., Niedobitek G., Crocker J., Brooks L., Rickinson A.B., Young L.S. (1993). Epstein-Barr virus and Hodgkin’s disease: Transcriptional analysis of virus latency in the malignant cells. J. Exp. Med..

[3] Niedobitek G., Agathanggelou A., Herbst H., Whitehead L., Wright D.H., Young L.S. (1997). Epstein-Barr virus (EBV) infection in infectious mononucleosis: Virus latency, replication and phenotype of EBV-infected cells. J. Pathol..

[4] Long H.M., Taylor G.S., Rickinson A.B. (2011). Immune defence against EBV and EBV-associated disease. Curr. Opin. Immunol..

[5] Veldman J., Visser L., Huberts-Kregel M., Muller N., Hepkema B., van den Berg A., Diepstra A. (2020). Rosetting T cells in Hodgkin lymphoma are activated by immunological synapse components HLA class II and CD58. Blood.

[6] Nijland M., Veenstra R.N., Visser L., Xu C., Kushekhar K., van Imhoff G.W., Kluin P.M., van den Berg A., Diepstra A. (2017). HLA dependent immune escape mechanisms in B-cell lymphomas: Implications for immune checkpoint inhibitor therapy?. Oncoimmunology.

[7] Diepstra A., van Imhoff G.W., Karim-Kos H.E., van den Berg A., te Meerman G.J., Niens M., Nolte I.M., Bastiaannet E., Schaapveld M., Vellenga E. (2007). HLA class II expression by Hodgkin Reed-Sternberg cells is an independent prognostic factor in classical Hodgkin’s lymphoma. J. Clin. Oncol..

[8] Sonmez M., Erkut N., Ucar F., Buruk K., Cobanoglu U., Bahce M., Ural A.U. (2010). Familial Hodgkin’s lymphoma from the perspective of HLA. Intern. Med..

[9] Diepstra A., Niens M., te Meerman G.J., Poppema S., van den Berg A. (2005). Genetic susceptibility to Hodgkin’s lymphoma associated with the human leukocyte antigen region. Eur. J. Haematol. Suppl..

[10] Diepstra A., Niens M., Vellenga E., van Imhoff G.W., Nolte I.M., Schaapveld M., van der Steege G., van den Berg A., Kibbelaar R.E., te Meerman G.J. (2005). Association with HLA class I in Epstein-Barr-virus-positive and with HLA class III in Epstein-Barr-virus-negative Hodgkin’s lymphoma. Lancet.

[11] Urayama K.Y., Jarrett R.F., Hjalgrim H., Diepstra A., Kamatani Y., Chabrier A., Gaborieau V., Boland A., Nieters A., Becker N. (2012). Genome-wide association study of classical Hodgkin lymphoma and Epstein-Barr virus status-defined subgroups. J. Natl. Cancer Inst..

[12] Cozen W., Li D., Best T., Van Den Berg D.J., Gourraud P., Cortessis V.K., Skol A.D., Mack T.M., Glaser S.L., Weiss L.M. (2012). A genome-wide meta-analysis of nodular sclerosing Hodgkin lymphoma identifies risk loci at 6p21.32. Blood.

[13] Frampton M., da Silva Filho M.I., Broderick P., Thomsen H., Forsti A., Vijayakrishnan J., Cooke R., Enciso-Mora V., Hoffmann P., Nothen M.M. (2013). Variation at 3p24.1 and 6q23.3 influences the risk of Hodgkin’s lymphoma. Nat. Commun..

[14] Cozen W., Timofeeva M.N., Li D., Diepstra A., Hazelett D., Delahaye-Sourdeix M., Edlund C.K., Franke L., Rostgaard K., Van Den Berg D.J. (2014). A meta-analysis of Hodgkin lymphoma reveals 19p13.3 TCF3 as a novel susceptibility locus. Nat. Commun..

[15] Niens M., Jarrett R.F., Hepkema B., Nolte I.M., Diepstra A., Platteel M., Kouprie N., Delury C.P., Gallagher A., Visser L. (2007). HLA-A*02 is associated with a reduced risk and HLA-A*01 with an increased risk of developing EBV+ Hodgkin lymphoma. Blood.

[16] Hjalgrim H., Rostgaard K., Johnson P.C., Lake A., Shield L., Little A.M., Ekstrom-Smedby K., Adami H.O., Glimelius B., Hamilton-Dutoit S. (2010). HLA-A alleles and infectious mononucleosis suggest a critical role for cytotoxic T-cell response in EBV-related Hodgkin lymphoma. Proc. Natl. Acad. Sci. USA.

[17] Huang X., Kushekhar K., Nolte I., Kooistra W., Visser L., Bouwman I., Kouprie N., Veenstra R., van Imhoff G., Olver B. (2012). HLA associations in classical Hodgkin lymphoma: EBV status matters. PLoS ONE.

[18] Johnson P.C., McAulay K.A., Montgomery D., Lake A., Shield L., Gallagher A., Little A.M., Shah A., Marsh S.G., Taylor G.M. (2015). Modeling HLA associations with EBV-positive and -negative Hodgkin lymphoma suggests distinct mechanisms in disease pathogenesis. Int. J. Cancer.

[19] Parham P., Ohta T. (1996). Population biology of antigen presentation by MHC class I molecules. Science.

[20] Liu Y., l Razak F.R., Terpstra M., Chan F.C., Saber A., Nijland M., van Imhoff G., Visser L., Gascoyne R., Steidl C. (2014). The mutational landscape of Hodgkin lymphoma cell lines determined by whole-exome sequencing. Leukemia.

[21] Wienand K., Chapuy B., Stewart C., Dunford A.J., Wu D., Kim J., Kamburov A., Wood T.R., Cader F.Z., Ducar M.D. (2019). Genomic analyses of flow-sorted Hodgkin Reed-Sternberg cells reveal complementary mechanisms of immune evasion. Blood Adv..

[22] Burr M.L., Sparbier C.E., Chan K.L., Chan Y.C., Kersbergen A., Lam E.Y.N., Azidis-Yates E., Vassiliadis D., Bell C.C., Gilan O. (2019). An evolutionarily conserved function of polycomb silences the MHC class I antigen presentation pathway and enables immune evasion in cancer. Cancer Cell.

[23] de Charette M., Houot R. (2018). Hide or defend, the two strategies of lymphoma immune evasion: Potential implications for immunotherapy. Haematologica.

[24] Wilkinson S.T., Vanpatten K.A., Fernandez D.R., Brunhoeber P., Garsha K.E., Glinsmann-Gibson B.J., Grogan T.M., Teruya-Feldstein J., Rimsza L.M. (2012). Partial plasma cell differentiation as a mechanism of lost major histocompatibility complex class II expression in diffuse large B-cell lymphoma. Blood.

[25] Jordanova E.S., Riemersma S.A., Philippo K., Giphart-Gassler M., Schuuring E., Kluin P.M. (2002). Hemizygous deletions in the HLA region account for loss of heterozygosity in the majority of diffuse large B-cell lymphomas of the testis and the central nervous system. Genes Chromosomes Cancer.

[26] Jordanova E.S., Philippo K., Giphart M.J., Schuuring E., Kluin P.M. (2003). Mutations in the HLA class II genes leading to loss of expression of HLA-DR and HLA-DQ in diffuse large B-cell lymphoma. Immunogenetics.

[27] Murray P.G., Constandinou C.M., Crocker J., Young L.S., Ambinder R.F. (1998). Analysis of major histocompatibility complex class I, TAP expression, and LMP2 epitope sequence in Epstein-Barr virus-positive Hodgkin’s disease. Blood.

[28] Lee S.P., Constandinou C.M., Thomas W.A., Croom-Carter D., Blake N.W., Murray P.G., Crocker J., Rickinson A.B. (1998). Antigen presenting phenotype of Hodgkin Reed-Sternberg cells: Analysis of the HLA class I processing pathway and the effects of interleukin-10 on epstein-barr virus-specific cytotoxic T-cell recognition. Blood.

[29] Oudejans J.J., Jiwa N.M., Kummer J.A., Horstman A., Vos W., Baak J.P., Kluin P.M., van der Valk P., Walboomers J.M., Meijer C.J. (1996). Analysis of major histocompatibility complex class I expression on Reed-Sternberg cells in relation to the cytotoxic T-cell response in Epstein-Barr virus-positive and -negative Hodgkin’s disease. Blood.

[30] Flavell K.J., Billingham L.J., Biddulph J.P., Gray L., Flavell J.R., Constandinou C.M., Young L.S., Murray P.G. (2003). The effect of Epstein-Barr virus status on outcome in age- and sex-defined subgroups of patients with advanced Hodgkin’s disease. Ann. Oncol..

[31] Rickinson A.B., Moss D.J. (1997). Human cytotoxic T lymphocyte responses to Epstein-Barr virus infection. Annu. Rev. Immunol..

[32] Brennan R.M., Burrows S.R. (2008). A mechanism for the HLA-A*01-associated risk for EBV+ Hodgkin lymphoma and infectious mononucleosis. Blood.

[33] Lee S.P., Thomas W.A., Murray R.J., Khanim F., Kaur S., Young L.S., Rowe M., Kurilla M., Rickinson A.B. (1993). HLA A2.1-restricted cytotoxic T cells recognizing a range of Epstein-Barr virus isolates through a defined epitope in latent membrane protein LMP2. J. Virol..

[34] Jones K., Wockner L., Brennan R.M., Keane C., Chattopadhyay P.K., Roederer M., Price D.A., Cole D.K., Hassan B., Beck K. (2016). The impact of HLA class I and EBV latency-II antigen-specific CD8(+) T cells on the pathogenesis of EBV(+) Hodgkin lymphoma. Clin. Exp. Immunol..

[35] Yao Y., Liu N., Zhou Z., Shi L. (2019). Influence of ERAP1 and ERAP2 gene polymorphisms on disease susceptibility in different populations. Hum. Immunol..

[36] Jiang P., Veenstra R.N., Seitz A., Nolte I.M., Hepkema B.G., Visser L., van den Berg A., Diepstra A. (2021). Interaction between ERAP alleles and HLA class I types support a role of antigen presentation in Hodgkin lymphoma development. Cancers.

[37] Praest P., Luteijn R.D., Brak-Boer I.G.J., Lanfermeijer J., Hoelen H., Ijgosse L., Costa A.I., Gorham R.D., Lebbink R.J., Wiertz E.J.H.J. (2018). The influence of TAP1 and TAP2 gene polymorphisms on TAP function and its inhibition by viral immune evasion proteins. Mol. Immunol..

[38] Diepstra A., Poppema S., Boot M., Visser L., Nolte I.M., Niens M., Te Meerman G.J., van den Berg A. (2008). HLA-G protein expression as a potential immune escape mechanism in classical Hodgkin’s lymphoma. Tissue Antigens.

[39] Rodgers J.R., Cook R.G. (2005). MHC class Ib molecules bridge innate and acquired immunity. Nat. Rev. Immunol..

