# Supplementary material for: HLA Expression in Relation to HLA Type in Classic Hodgkin Lymphoma Patients
Source: Cancers (Basel). 2021 Nov 20;13(22):5833. doi: 10.3390/cancers13225833 (PMC8616181; doi:10.3390/cancers13225833)
Supplement: Supplementary file 1 [file cancers-13-05833-s001.zip › cancers-1410931-supplementary.pdf]

cancers

MDPI

Supplementary Materials: HLA Expression in Relation to HLA Type in Classic Hodgkin Lymphoma Patients

Geok Wee Tan, Peijia Jiang, Ilja M. Nolte, Kushi Kushekhar, Rianne N. Veenstra, Bouke G. Hepkema, Ruth F. Jarrett, Anke van den Berg and Arjan Diepstra

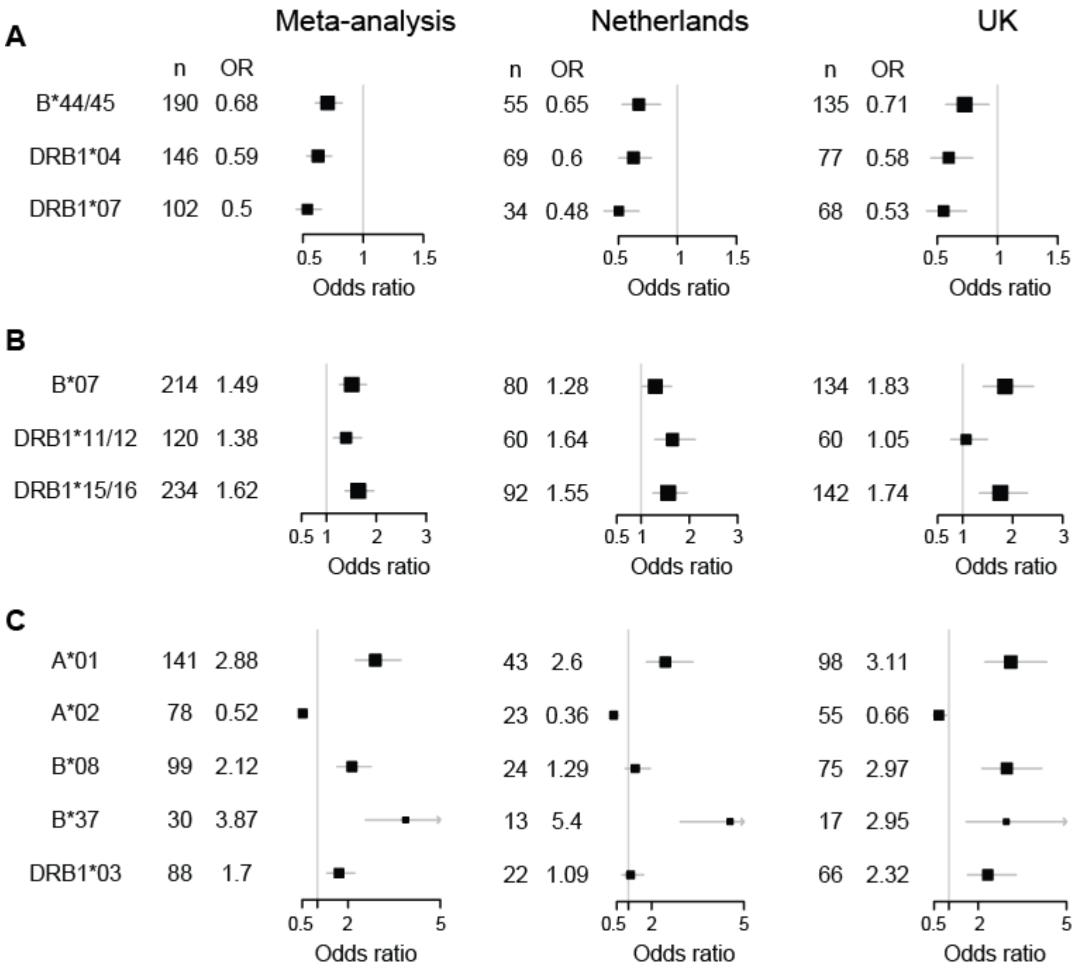

Figure S1. Forest plots of significant cHL susceptibility or protective HLA alleles. Significant cHL-associated HLA alleles ($p < 0.01$) in meta-analysis, Netherlands cohort, and UK cohort for (**A**) cHL overall, (**B**) EBV- and (**C**) EBV+. Odds ratios with 95% confidence interval (CI) are shown and arrows represent clipping of lower or upper limits for CI.

Table S1. Number of subjects in meta-analysis and HLA expression analysis.

	Netherlands	UK	Combination
	n **(%)**	***n*** **(%)**	***n*** **(%)**
**Meta-analysis**			
Patients	337	502	839
EBV-	232 (68.8%)	348 (69.3%)	580 (69.1%)
EBV+	78 (23.1%)	154 (30.7%)	232 (27.7%)
EBV unknown	27 (8.0%)	0 (0%)	27 (3.2%)
Controls	347	7901	
**HLA expression analysis**			
Patients	254	84	338
EBV-	193 (76.0%)	57 (67.9%)	250 (74.0%)
EBV+	59 (23.2%)	27 (32.1%)	86 (25.4%)
EBV unknown	2 (0.8%)	0 (0%)	2 (0.6%)

Table S2. Frequencies of HLA alleles in patients and controls that were previously found to be suggestive ($p < 0.10$) in either cohort.

	**Netherlands**								**UK**							
	**EBV+ cases**		**EBV− cases**		**All cases**		**Controls**		**EBV+ cases**		**EBV− cases**		**All cases**		**Controls**	
HLA-A	78		232		337		7554		154		348		502		347	
A1	43	55%	70	30%	120	36%	2426	32%	98	64%	123	35%	221	44%	125	36%
A2	23	29%	123	53%	162	48%	4052	54%	55	36%	173	50%	228	45%	158	46%
A3	27	35%	56	24%	94	28%	2296	30%	34	22%	87	25%	121	24%	99	29%
A11	8	10%	30	13%	38	11%	713	9%	11	7%	37	11%	48	10%	35	10%
A19	12	15%	38	16%	55	16%	1620	21%	29	18%	77	22%	106	21%	76	22%
A28	4	5%	26	11%	33	10%	607	8%	7	5%	22	13%	29	6%	29	8%
HLA-B	78		229		333		7554		154		346		500		347	
B5	13	17%	33	14%	51	15%	784	10%	28	18%	10	3%	38	8%	26	8%
B7	25	32%	80	35%	115	35%	2233	30%	29	19%	134	39%	163	33%	89	26%
B8	24	31%	59	26%	88	26%	1932	26%	75	49%	97	28%	172	34%	84	24%
B12	12	15%	39	17%	55	17%	1765	23%	39	16%	96	21%	135	27%	119	34%
B15	10	13%	47	21%	61	18%	1434	19%	10	6%	50	14%	60	12%	35	10%
B27	1	1%	16	7%	18	5%	603	8%	6	4%	35	10%	41	8%	38	11%
B37	13	17%	10	4%	23	7%	270	4%	17	11%	11	3%	28	6%	14	4%
B40	13	17%	26	11%	44	13%	1339	18%	31	20%	27	8%	58	12%	32	9%
HLA-DR	78		231		336		6559		144		325		469		311	
DR2	25	32%	92	40%	127	38%	1967	30%	25	17%	142	44%	167	36%	96	31%
DR3	22	28%	55	24%	84	25%	1734	26%	66	46%	99	30%	165	35%	83	27%
DR4	23	29%	43	19%	69	21%	1969	30%	20	14%	57	18%	77	16%	79	25%
DR5	8	10%	60	26%	79	24%	1158	18%	12	8%	60	18%	72	15%	55	18%
DR6	28	36%	75	32%	113	34%	1989	30%	21	15%	48	15%	69	15%	32	10%
DR7	7	9%	25	11%	34	10%	1246	19%	26	18%	42	13%	68	14%	75	24%
